# Technology-Enhanced Exercise Training for Cardiometabolic Syndrome: A Scoping Review

**DOI:** 10.3390/jfmk11020153

**Published:** 2026-04-14

**Authors:** Iosif-Alexandros Kouidis, Pantazis Deligiannis, Anastasia Theofanous, Maria Anifanti, Evangelia Kouidi

**Affiliations:** 1 Laboratory of Sports Medicine, Department of Physical Education and Sports Science, Aristotle University of Thessaloniki, 57001 Thessaloniki, Greece; joskouidi@gmail.com (I.-A.K.); maynyfant@phed.auth.gr (M.A.); 2 Department of Medicine, School of Medicine, European University Cyprus, Egkomi, 2404 Nicosia, Cyprus; siapafos@gmail.com; 3 Microsoft Research, 14820 NE 36th St., Redmond, WA 98052, USA; pdeligia@me.com

**Keywords:** metabolic syndrome, cardiometabolic risk, exercise training, structured physical activity, wearable devices, telemonitoring, artificial intelligence, virtual reality, exergaming, continuous glucose monitoring

## Abstract

**Background**: Μetabolic syndrome (MetS)—comprises central adiposity, elevated blood pressure, dyslipidaemia, and dysglycaemia, increasing the risk of type 2 diabetes and cardiovascular disease. Exercise training improves cardiorespiratory fitness and several MetS components, but real-world effectiveness is limited by poor adherence, restricted supervision, and insufficient personalisation. **Objective:** This scoping review mapped the clinical intervention evidence on technology-enhanced exercise and structured physical activity relevant to MetS, while distinguishing direct MetS evidence from translational evidence. **Methods**: In accordance with PRISMA-ScR, we searched PubMed and extended the search to Scopus and Web of Science; a supplementary IEEE Xplore search and a post hoc Embase check were also conducted. Eligible studies were interventions using web-based delivery, wearables, telemonitoring/mobile health (mHealth), artificial intelligence (AI) coaching, virtual reality (VR)/exergaming, or continuous glucose monitoring (CGM) alongside exercise training or structured physical activity. **Results**: Nineteen studies met the eligibility criteria. The evidence base was weighted toward wearable/app-based feedback and telemonitoring/mHealth/web-based approaches, with fewer studies on VR/exergaming, CGM-enabled exercise, and AI coaching. Most studies were randomised or cluster-randomised, but interventions were usually short term. Across categories, technology most consistently supported adherence, self-monitoring, accountability, remote supervision, and, in selected cases, physiology-informed personalisation. Direct MetS evidence was strongest for wearables with structured feedback, telemonitoring, mHealth, and web-based delivery, whereas AI coaching and CGM were supported by adjacent translational evidence. **Conclusions**: Technology-enhanced exercise and structured physical activity show promising but heterogeneous and still preliminary potential for MetS management. Key limitations include short follow-up, uneven representation across categories, inconsistent reporting of exercise dose/intensity fidelity and adverse events, and limited equity and implementation outcomes.

## 1. Introduction

Cardiometabolic syndrome, also known as metabolic syndrome (MetS), comprises central obesity, high blood pressure, abnormal cholesterol levels, and impaired blood sugar control. Together, these factors increase the risk of type 2 diabetes and heart disease [1,2,3]. Although standard criteria have helped define MetS, it still varies widely between individuals, particularly in insulin resistance, fat distribution, and responses to lifestyle changes [1,2,3]. Exercise is a key treatment. It boosts cardiorespiratory fitness (CRF), reduces abdominal fat, improves muscle glucose uptake, and can improve blood pressure and cholesterol [4,5]. People with higher CRF have fewer heart problems and a lower risk of death from any cause. This makes exercise central to preventing and treating cardiometabolic problems [6].

Although exercise interventions show success in experimental settings, real-world effectiveness is often limited by poor adherence, restricted access to supervised programmes, and difficulties in verifying training dose and intensity in free-living settings. Emerging technologies, including wearable sensors, mobile health applications, and remote monitoring platforms, can help address this efficacy–effectiveness gap by providing real-time feedback, enabling remote supervision, personalising exercise prescriptions through physiological biofeedback, and supporting sustained behaviour change with actionable data. These technologies should not be viewed as substitutes for exercise itself, but rather as delivery tools that may improve how exercise or structured physical activity is implemented, monitored, and sustained in everyday life.

At the same time, the available evidence is heterogeneous. Technology-enabled interventions differ substantially in target population, behavioural intensity, degree of supervision, comparator conditions, and the specific role of technology itself, whether for self-monitoring, feedback, accountability, remote support, or physiological personalisation. Moreover, while MetS is the primary clinical focus, adjacent cardiometabolic phenotypes such as prediabetes, overweight/obesity, impaired glucose regulation, and type 2 diabetes may also be informative when the intervention or outcomes map directly onto MetS components. This is especially important for emerging approaches such as CGM-guided exercise personalisation, where translational evidence may first appear in dysglycaemic populations before being tested directly in MetS-defined cohorts.

Against this background, the aim of the present scoping review was to systematically map clinical intervention evidence on technology-enhanced exercise and structured physical activity interventions relevant to MetS and closely aligned cardiometabolic phenotypes. Specifically, we sought to examine how different technology-enhanced interventions—wearables, telemonitoring/mHealth, AI coaching, VR/exergaming, and CGM—may support adherence, remote supervision, and personalised exercise delivery, while explicitly distinguishing direct MetS evidence from adjacent translational evidence and identifying key evidence gaps related to study design, reporting quality, long-term maintenance, and clinical translation.

## 2. Materials and Methods

This scoping review was conducted and reported in accordance with the PRISMA extension for Scoping Reviews (PRISMA-ScR) [7]. A completed PRISMA-ScR checklist (Table S1) and the corresponding flow diagram (Figure S1) are provided in the Supplementary Materials. The review protocol was not prospectively registered.

### 2.1. Information Sources and Search Strategy

The primary database workflow comprised PubMed, Scopus, and Web of Science and was designed to reduce database bias and improve coverage of technology-mediated exercise and structured physical activity interventions. A supplementary search of IEEE Xplore was also undertaken to improve capture of technology-oriented literature. PubMed was searched in January 2026, and Scopus, Web of Science, and IEEE Xplore were searched in March 2026. Search terms combined: (i) cardiometabolic phenotypes (metabolic syndrome/cardiometabolic syndrome and, where analytically relevant to MetS components, prediabetes, overweight/obesity, impaired glucose regulation, and type 2 diabetes for CGM-guided personalisation), (ii) exercise training/structured physical activity, and (iii) enabling technologies (web-based or internet-delivered interventions, wearables/activity trackers, telemonitoring/remote monitoring, mHealth/mobile apps, AI/digital coaching, virtual reality/exergaming, and continuous glucose monitoring). Across databases, the search strategy combined free-text terms and, where available, controlled vocabulary. In PubMed, controlled vocabulary terms (e.g., MeSH) were used where relevant, while database-adapted keyword-based syntax and field tags were applied in Scopus and Web of Science. The final database-specific search strings and yields are reported in Supplementary Material S1.

The yields for the primary database were PubMed (*n* = 582), Scopus (*n* = 27), and Web of Science (*n* = 29). Records from the primary databases were exported to a reference manager for cross-database deduplication prior to final screening and full-text assessment. The expansion of the search beyond PubMed primarily increased coverage of web-based, telemonitoring, and mHealth-oriented interventions. The supplementary IEEE Xplore search did not contribute additional eligible clinical intervention studies. In addition, a post hoc supplementary Embase check was undertaken during manuscript refinement after institutional access limitations had initially prevented Embase from being included in the primary search workflow. This supplementary check identified one additional eligible study, which was assessed against the prespecified eligibility criteria and incorporated into the final evidence map. Accordingly, the final evidence map reflects a transparently reported mixed search process rather than a single prospectively defined workflow, and Figure S1 reports the final counts from the updated reference-management workflow.

### 2.2. Eligibility Criteria

Eligible sources of evidence were adult clinical intervention studies that incorporated at least one of the above technologies alongside an exercise training or structured physical activity intervention. This included web-based or internet-delivered support, telemonitoring, mobile health, wearables, AI coaching, VR/exergaming, and CGM-enabled approaches when these were used to deliver, monitor, personalise, or support exercise or structured physical activity. Included designs comprised randomised controlled trials, non-randomised controlled trials, pilot trials, and prospective intervention studies (including quasi-experimental and observational intervention designs). Studies in MetS-defined cohorts were prioritised as direct MetS evidence. Studies in closely aligned cardiometabolic phenotypes (e.g., prediabetes/overweight or obesity, impaired glucose regulation, and type 2 diabetes) were included when outcomes were directly relevant to MetS components (e.g., cardiorespiratory fitness, anthropometrics, blood pressure, glycaemic indices, lipids, or composite metabolic risk) or when the phenotype was specifically relevant to physiology-guided exercise personalisation, as in CGM-based exercise timing in dysglycaemic populations. This approach was used to map the translational boundary of the field while preserving MetS as the primary clinical anchor of the review.

Review articles, study protocols without outcome data, and case reports/series were excluded. Outcomes charted included physical activity/adherence, CRF/fitness, anthropometrics (waist, body fat, visceral fat), blood pressure, glycaemic indices (fasting glucose, HbA1c, and CGM metrics), lipids, and composite MetS severity, when available.

### 2.3. Selection of Sources of Evidence and Data Charting

Titles/abstracts were screened for relevance, and full texts were assessed for all reports retained for inclusion. Data were charted using a prespecified extraction framework capturing population, design/duration, technology function, and key outcomes. Two reviewers independently screened titles/abstracts and assessed full texts for eligibility. Discrepancies were resolved by discussion and, when needed, consultation with a third reviewer. Data charting was performed using a prespecified extraction form; a second reviewer verified extracted data for completeness and accuracy. Results were synthesised descriptively and mapped in an evidence table. As this was a scoping review, a formal risk-of-bias assessment was not used to exclude studies; however, we systematically charted design features that influence internal validity and implementation interpretability (randomisation, comparator type, follow-up duration, attrition reporting, adverse-event reporting, and whether exercise dose/intensity was objectively verified). This additional charting was undertaken to support a more analytic discussion of why reported effects may differ across technology categories despite heterogeneous interventions and outcomes.

### 2.4. Data Synthesis and Analysis

Given the scoping nature of the review and the substantial heterogeneity across populations, interventions, outcomes, and follow-up, no meta-analysis or hypothesis testing was performed. Study characteristics and outcomes were summarised using descriptive statistics (counts and percentages) and, where applicable, the median and range for continuous variables such as intervention duration and sample size. Studies and outcomes were mapped by technology category and cardiometabolic phenotype, and the direction of effect was narratively synthesised across MetS components and related endpoints without statistical pooling. Attention was paid to whether technology functioned primarily as a monitoring tool, a feedback/behaviour-change tool, a supervision layer, or a physiological personalisation tool. Descriptive analyses were performed in Microsoft Excel (Microsoft Corporation, Redmond, WA, USA). Study characteristics and outcomes were summarised using descriptive statistics (counts and percentages) and, where applicable, the median and range for continuous variables such as intervention duration and sample size. No inferential statistical analyses were performed.

## 3. Results

### 3.1. Overview of the Evidence Base and Contextual Exercise Evidence

To contextualise why delivery fidelity matters, foundational clinical exercise evidence in adults with MetS shows that aerobic interval training can improve CRF more than continuous moderate training and can reduce the number of MetS risk factors [8]. Independent evidence also indicates that higher-intensity training preferentially reduces visceral abdominal fat, even when energy expenditure is controlled [9]. Modality comparisons show distinct adaptations: aerobic training is especially effective for reducing fat mass, resistance training supports lean mass, and combined training offers broad benefits but may not always exceed aerobic training for adiposity outcomes [10,11]. Longer-duration pragmatic trials (e.g., CardioRACE) support sustained training effects on composite cardiovascular risk profiles in overweight/obese adults with elevated blood pressure [12]. In MetS, HIIT (high-intensity interval training) and moderate continuous training can both improve metabolic outcomes, provided feasibility and safety are carefully considered [13,14]. These foundational exercise trials provide contextual background for technology-enabled delivery but were not part of the technology-enabled evidence map. After completion of the primary database workflow (PubMed, Scopus, and Web of Science) and the supplementary Embase check, nineteen clinical intervention studies met the eligibility criteria and were included in the final evidence map (Table 1). The evidence base included direct MetS studies as well as adjacent translational studies in closely related cardiometabolic phenotypes when outcomes mapped directly onto MetS components or informed physiology-guided exercise personalization. Three studies identified through Scopus/Web of Science strengthened the telemonitoring/mHealth category [15,16,17], and the supplementary Embase check identified one additional eligible web-based intervention study [18]. The supplementary IEEE Xplore search did not contribute additional eligible intervention studies.

### 3.2. Wearables and App-Based Feedback

In people with MetS, using wearables to provide feedback increased physical activity and improved some MetS measures [19,20]. Table 1 presents the final evidence map across MetS-defined cohorts and adjacent cardiometabolic phenotypes included as translational evidence. Wearables and app-based programmes have also been tested in groups at risk of MetS and in young adults [21,22]. Wearables have been used to help people maintain physical activity after structured exercise programmes, particularly among employees with MetS, but sustaining activity levels remains difficult even when some health benefits persist [23]. Overall, wearable interventions appeared most useful when monitoring data were translated into actionable goals, feedback, and accountability rather than used for passive self-tracking alone.

### 3.3. Telemonitoring, Mobile Health, and Web-Based Delivery

An earlier internet-delivered physical activity intervention in adults with MetS supported the feasibility and acceptability of web-based delivery, suggesting that digitally mediated exercise support in MetS predates more recent app- and wearable-based models [18]. Workplace telemonitoring interventions that support physical activity have been found to reduce MetS severity and improve work ability [4]. Year-long telemonitoring of nutrition and activity has resulted in significant weight loss and improvements in MetS markers [24]. Additional mobile health studies have evaluated the integration of health apps with exercise plans, aiming to reduce cardiometabolic risk and support weight management in individuals with MetS [25,26]. Three studies identified through Scopus and Web of Science further strengthened this category, including smart-device self-management, individualized WeChat-based follow-up, and IVRS-supported mHealth delivery [15,16,17]. Collectively, telemonitoring, mHealth, and web-based delivery approaches can support structured physical activity and broader lifestyle execution in MetS, although the independent contribution of the technology remains difficult to separate from programme intensity and behavioural support.

### 3.4. AI (Artificial Intelligence) Coaching

Evidence for AI coaching remained limited but clinically relevant. A clinical trial included in the final evidence map found that a fully automated, AI-powered Diabetes Prevention Program was as effective as human coaching in improving weight, HbA1c, and physical activity among adults with prediabetes and those who were overweight or obese [27]. Because this evidence derives from an adjacent high-risk phenotype rather than a MetS-defined cohort, AI coaching should currently be interpreted as promising translational evidence rather than established direct MetS evidence.

### 3.5. VR (Virtual Reality) and Exergaming

A randomized trial found that immersive VR exergames with adaptive resistance improved fitness and cardiometabolic health more than standard approaches [28]. In individuals with MetS, another exergaming trial enhanced executive function and demonstrated the feasibility of this method [29]. Although few in number, these studies suggest that immersive and game-based approaches may be particularly relevant when enjoyment, motivation, or cognitive engagement are major barriers to sustained participation.

### 3.6. CGM (Continuous Glucose Monitoring)-Enabled Approaches

Real-time feedback from CGM has helped individuals with poor blood sugar control adhere to exercise regimens [30]. CGM has also been used to personalise exercise for people with type 2 diabetes, targeting periods of peak blood sugar to improve glucose and vascular health. This evidence supports similar methods for MetS patients with blood sugar challenges [31]. Using CGM, with or without activity trackers, has been shown to improve blood sugar control, indicating that combining these tools may help people remain physically active [32]. Overall, CGM-enabled approaches were studied mainly in dysglycaemic phenotypes rather than in MetS-defined cohorts. Their relevance to MetS should therefore be interpreted as translational and promising, not as directly established MetS-specific evidence.

### 3.7. Cross-Cutting Implementation Considerations

Across technologies, feasibility and clinical impact were highest when (i) objective dose metrics were combined with structured feedback (such as goal-setting and accountability), (ii) intensity fidelity was verified (for example, using heart-rate zones instead of step counts alone), and (iii) the technology stack was embedded in a behaviour-change pathway that supports progression and maintenance [4,16,17,18,19,23,24,30,31]. However, reporting of adverse events, equity-related barriers (such as digital literacy and device cost), and data governance was inconsistent across trials, representing a significant gap for translation into routine care.

### 3.8. Descriptive and Analytic Synthesis Across the Evidence Map

Across the 19 included intervention studies (Table 1), the evidence base was weighted toward wearable/app-based feedback and telemonitoring/mHealth/web-based models (13/19 in total; wearable/app-based feedback, 5/19; telemonitoring/mHealth/web-based delivery, 8/19), with fewer studies evaluating CGM-enabled exercise approaches (3/19), VR/exergaming (2/19), and AI coaching (1/19). MetS-defined cohorts accounted for a substantial proportion of the literature, while the remaining studies examined closely related cardiometabolic phenotypes including MetS risk/prevention, metabolic risk, impaired glucose regulation, prediabetes with overweight/obesity, and type 2 diabetes for CGM-guided exercise personalisation. Most studies were randomised or cluster-randomised trials, while the remaining evidence derived from pilot, quasi-experimental, observational intervention designs. Where duration was reported, interventions were most commonly short term (~8–12 weeks), with relatively few maintenance-phase or longer-term studies.

Interpretability of cardiometabolic effects depended strongly on whether the technology verified training dose and intensity (e.g., heart-rate zone time, session completion, or CGM-derived physiological response) rather than relying mainly on step-count or self-report proxies. Studies that combined objective monitoring with structured feedback loops (goal setting, accountability, escalation when targets were missed, and/or periodic human contact) more consistently reported improvements in downstream MetS-related outcomes than those focused primarily on self-monitoring alone. Where comparators were not attention matched, some observed improvements may also reflect non-specific support effects such as contact frequency, coaching time, or accountability.

Maintenance after the active support phase was assessed inconsistently, and when reported, sustained physical activity was often harder to preserve than short-term risk-factor improvements. In addition, only a minority of studies reported adverse events in a structured way, and equity-related barriers (device cost, digital literacy, language accessibility, and connectivity) were rarely operationalized as outcomes. These limitations reduce the certainty of cross-category comparisons and constrain translation to routine cardiometabolic care pathways.

**Figure 1:**
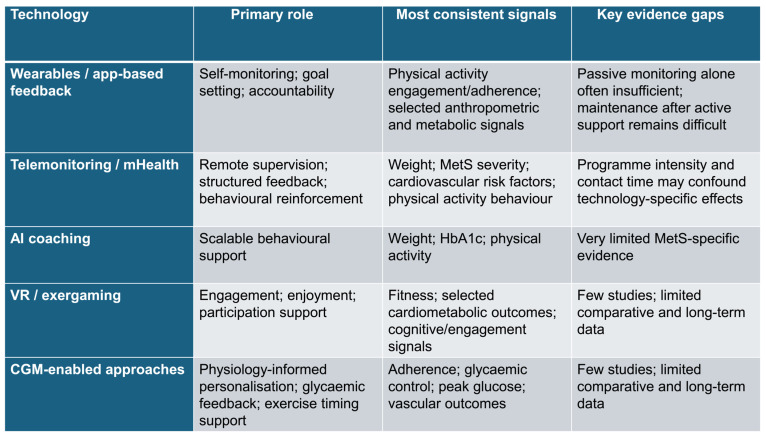
Evidence-based conceptual framework of technology-enhanced exercise and structured physical activity interventions in cardiometabolic syndrome and related cardiometabolic phenotypes. The figure summarises the major technology categories identified in the evidence map, their primary role within interventions, the outcomes most consistently supported, and the main limitations of the current evidence base. Where relevant, the framework distinguishes between direct MetS evidence and adjacent translational evidence. Wearables/app-based feedback and telemonitoring/mHealth are the most represented categories, whereas AI coaching, VR/exergaming, and CGM-enabled approaches remain less extensively studied.

Figure 1 presents an evidence-based conceptual framework derived from the mapped studies, illustrating the primary role of each technology category, the outcomes most consistently supported, and the major limitations affecting interpretation and translation. Where relevant, the framework clearly distinguishes between direct MetS evidence and adjacent translational evidence.

## 4. Discussion

Across the 19 included clinical intervention studies, technology most consistently appeared to add value by improving process determinants of exercise and structured physical activity delivery rather than by demonstrating uniform superiority of any single platform. In practical terms, the most reproducible contribution of technology was to support self-monitoring, goal pursuit, accountability, remote supervision, engagement, and, in selected cases, physiology-informed personalisation. This interpretation aligns with foundational MetS trials showing that intensity and delivery fidelity strongly shape outcomes [8,9,13,14].

For interpretive clarity, direct MetS evidence in this review refers to studies enrolling MetS-defined cohorts, whereas adjacent translational evidence refers to closely related phenotypes included only when outcomes mapped directly onto MetS components or informed physiology-guided exercise personalization. Maintaining this distinction is essential to avoid overextending conclusions beyond the available evidence base. The strongest evidence for MetS to date comes from studies using wearables and telemonitoring. Trials with wearables show that turning sensor data into feedback, such as setting goals, giving advice, and keeping people accountable, can improve activity and some MetS measures [19,20,21,22,23]. An earlier internet-delivered physical activity intervention in adults with MetS also supported the feasibility and acceptability of web-based delivery, suggesting that digitally mediated exercise support in MetS predates more recent app- and wearable-based models [18]. Telemonitoring is also very useful, with one workplace study showing reduced MetS severity [4] and a year-long trial showing real weight loss and improved MetS markers [24]. Even in these categories, however, the incremental contribution of technology is difficult to separate from the behavioral program wrapped around it.

Beyond the 19 technology-enabled intervention studies mapped in Table 1, additional pragmatic evidence from related digital health interventions helps clarify which components drive benefit. In a 12-week randomized trial in free-living sedentary adults, a multicomponent approach combining a consumer wearable activity tracker with motivational interviewing reduced sedentary time and improved several cardiometabolic variables, whereas self-monitoring with the tracker alone showed little effect supporting the view that feedback structure and human support (even if brief) may be necessary to translate sensor data into clinically meaningful change [33]. In underserved populations, a pilot randomised trial using Fitbit-based monitoring plus periodic individualised counselling suggested that benefits may be greatest among participants with low baseline activity, highlighting the need to tailor support intensity to initial risk and digital readiness [34]. At the systems level, a large population-based cohort within a national mHealth program reported that both wearables and smartphone-built-in step counters improved walking behaviours and reduced metabolic syndrome risk, suggesting that lower-cost device options may deliver comparable population benefit when embedded in an organised service pathway [35].

### 4.1. Lifestyle Effects Beyond Exercise: Sleep, Wake-Up Regularity, and Dietary Behaviour

The present review does not support a strong MetS-specific conclusion regarding sleep timing, wake-up regularity, or sleep duration because these outcomes were not reported consistently in the included intervention studies. Most studies focused primarily on physical activity, fitness, weight-related outcomes, glycemic measures, or composite MetS markers.

Several telemonitoring and mHealth interventions were multi-behaviour lifestyle programs in which physical activity was an explicit target but diet, self-management routines, or broader health behaviors were also addressed [15,16,17,24,25,26]. Accordingly, the most accurate interpretation is that current MetS-relevant evidence supports technology as a facilitator of physical activity execution and broader lifestyle self-management, but not yet as a clearly established modifier of sleep-related routines within this specific literature.

### 4.2. Mental State, Motivation, and Psychological Engagement

Direct mental-state outcomes were rarely measured in the included studies, limiting the strength of the conclusions. Nevertheless, the mapped evidence suggests that technology may influence psychological determinants of exercise adherence. For example, exergaming in MetS improved executive function and demonstrated feasibility [29], while other technology-supported models appeared to enhance engagement and perceived structure during exercise [15,16,17,18,19,20,21,22,23,24,25,26]. These findings are best interpreted as signals that digital features may affect mediators of adherence—such as self-efficacy, perceived competence, motivation, or burden—rather than as consistently demonstrated clinical mental-health outcomes within the current MetS evidence base.

### 4.3. Do Effects Differ Between Lean and Obese Individuals?

The present review cannot provide a robust answer to whether wearable devices, structured feedback, or telemonitoring produce different positive health effects in lean versus obese individuals. Most included studies enrolled participants with MetS, overweight/obesity, prediabetes, metabolic risk, or type 2 diabetes rather than explicitly comparing responses across adiposity strata [15,21,22,25,27,30,31,32].

A more defensible interpretation is that the absolute clinical benefit of technology-enhanced exercise may be greater in those with higher baseline cardiometabolic risk, more adiposity, lower initial physical activity, or poorer glycaemic control, because these participants have greater room for improvement. However, this should not be overstated as proof that the technology itself works differently in lean versus obese individuals.

### 4.4. Accessibility, Suitability, and Digital Inequity

For scalable adoption, interventions should explicitly describe the digital behaviour-change strategy (who monitors data, how feedback is delivered, and how nonadherence triggers escalation). Higher-intensity prescriptions may require screening and staged progression, particularly in older adults or those with multimorbidity. However, wearable devices, structured feedback, and telemonitoring are not equally accessible to everyone. Device cost, smartphone ownership, internet access, language, digital literacy, sensory or cognitive limitations, and willingness to engage with self-monitoring can all determine whether a participant is realistically able to benefit from a digital intervention. Yet such barriers were rarely measured as outcomes in the included studies [15,16,17,19,20,21,22,23,24,25,26,30,31,32].

Therefore, technology-enhanced exercise should not be presented as universally scalable in its current form. A more realistic conclusion is that these tools are promising, but their successful implementation depends on matching the technological burden to the patient’s digital readiness, resources, and support needs.

### 4.5. Mechanistic Interpretation: Why Weight, Visceral Fat, and Glycaemia May Improve

Although MetS-defined cohorts were prioritised, evidence from closely related cardiometabolic phenotypes was included only when outcomes were directly relevant to MetS components or when they informed physiology-guided exercise personalization. Accordingly, AI coaching and CGM should currently be interpreted as promising translational strategies for MetS rather than directly established MetS-specific interventions. A mechanistic interpretation is also needed to explain why these technologies may contribute to weight loss, reductions in visceral adiposity, and improved blood glucose. The most important point is that the mechanism is likely behavioural first and physiological second. Technology does not replace exercise physiology; rather, it increases the likelihood that the intended exercise stimulus is delivered with sufficient dose, consistency, and fidelity.

Weight loss is therefore most plausibly explained by improved adherence to physical activity goals, greater weekly activity volume, reduced inactivity, and in some programmes, concurrent improvements in diet or broader lifestyle self-management [15,16,17,24,25,26]. Similarly, reductions in visceral fat are most likely to occur when technology helps participants reach and sustain effective exercise intensity and progression. Improved glycaemic control can be understood through established exercise-mediated mechanisms, including increased skeletal muscle glucose uptake, improved insulin sensitivity, and reduced postprandial glucose excursions. CGM-enabled approaches add a more specific physiological advantage: they can make glucose variability visible to the patient, reinforce adherence through immediate biofeedback, and help align exercise timing with periods of peak glycaemic vulnerability [30,31,32].

### 4.6. A More Cautious Interpretation of What Technology Adds Beyond a Good Exercise Prescription

Recent perspectives on publication highlight that landmark 2025 trials—largely driven by new pharmacotherapies for diabetes and MetS with metabolic and renal benefits—are reshaping cardiovascular risk and reducing mortality [36]. However, for the purposes of the present review, the central clinical question is not whether exercise benefits MetS—this is well established—but what incremental value technology provides beyond a well-designed exercise prescription delivered with standard counselling. Across the mapped evidence, technology appears most valuable when it reduces uncertainty about what was completed, improves fidelity to intended exercise dose and intensity, and creates rapid feedback cycles that support progression and adherence. Conversely, when interventions rely mainly on self-monitoring without structured feedback or escalation, benefits are less consistent and may not exceed those achievable with conventional behavioural support.

Important gaps in the evidence include longer follow-up to assess whether benefits persist, standardised measures of MetS severity, direct comparisons of technologies with equal support time, and practical studies across diverse groups to ensure universal access. The present evidence does not yet justify a definitive stepped-care pathway, nor does it allow firm conclusions about which technology tier is sufficient for which patient profile; at most, the current literature supports this as a hypothesis for future head-to-head testing with matched support time.

Although not part of the mapped intervention evidence base, broader evidence syntheses from adjacent digital-health fields help contextualize the present findings. Systematic reviews and meta-analyses suggest that wearable physical activity trackers and activity monitors can improve physical activity and selected cardiometabolic outcomes, particularly when combined with behaviour-change support rather than passive self-monitoring alone [37,38]. Similarly, eHealth interventions appear to support weight loss and weight-loss maintenance in adults with overweight or obesity [39], while internet-based smartphone applications have also shown consistent benefit for improving healthy eating behaviours [40]. At the same time, implementation and scalability depend not only on efficacy but also on user acceptance: patient and public attitudes toward health monitoring technologies are shaped by perceived usefulness, burden, trust, privacy, and contextual fit [41]. This broader perspective is especially relevant because sleep is increasingly recognised as an important determinant of cardiometabolic health [42], and mHealth applications targeting sleep already incorporate behaviour change techniques in structured ways [43].

### 4.7. Reporting Quality, Implementation, and Research Priorities

Digital exercise interventions are often described at a high level, yet small design choices (feedback frequency, escalation rules, coaching content, and data thresholds for progression) can materially change adherence and outcomes. To improve interpretability and replication, future technology-enabled exercise trials should follow eHealth-specific reporting guidance (CONSORT-eHEALTH: Consolidated Standards of Reporting Trials—eHEALTH) and provide a complete, structured description of intervention components (TIDieR: Template for Intervention Description and Replication), including the “active ingredients” of behaviour change, fidelity checks, and the exact data streams used to trigger tailored messaging or clinician contact [44,45].

Beyond statistically significant effects, future studies should also report pragmatic implementation outcomes such as reach, adoption, implementation fidelity, and maintenance. Applying implementation-evaluation frameworks such as RE-AIM can clarify who benefits, whether the intervention is deliverable in routine care, and whether behaviour change is sustained after active support ends [46]. In addition, given that some technology stacks are intended to support progression toward higher-intensity prescriptions, explicit and evidence-informed preparticipation screening and staged progression remain important to maximise safety while minimising unnecessary barriers to participation [47].

Accordingly, future MetS-oriented trials should prioritise head-to-head comparisons with matched support time, clearer reporting of behaviour-change components and escalation rules, explicit implementation outcomes, and stratified analyses to determine which patients are likely to benefit from lower-burden self-monitoring versus more intensive telemonitoring or physiology-guided personalisation.

#### Limitations

This scoping review provides a narrative evidence map rather than a quantitative synthesis; although the primary search was expanded beyond PubMed to include Scopus and Web of Science, and a supplementary IEEE Xplore search was also undertaken, a post hoc Embase check during manuscript refinement identified one additional eligible study. Consequently, the final evidence map reflects a transparently reported mixed search process rather than a single prospectively defined workflow. Other potentially relevant databases and engineering-focused sources were not searched systematically, so some technology-oriented studies may still have been missed. The review protocol was not prospectively registered. The included studies varied substantially in phenotype definition, intervention design, technology function, comparator intensity, follow-up duration, and outcome reporting, limiting direct cross-category comparisons.

Formal risk-of-bias assessment was not used as an exclusion criterion; instead, design features relevant to internal validity and implementation interpretability were systematically charted to contextualise the findings. Finally, some technologies—particularly AI coaching, VR/exergaming, and CGM-enabled exercise personalisation—were represented by relatively few studies, so conclusions for these categories remain exploratory.

## 5. Conclusions

Technology-enhanced exercise and structured physical activity interventions show promising—but still heterogeneous and mostly preliminary—potential for the management of cardiometabolic syndrome and related high-risk phenotypes. Evidence in MetS-defined cohorts is strongest for wearables with structured feedback, telemonitoring, mHealth, and web-based delivery. AI coaching and CGM are supported primarily by adjacent high-risk or dysglycaemic phenotypes and should currently be interpreted as promising translational approaches for future MetS-specific trials rather than as directly established MetS interventions. Broader translation to MetS care will require longer follow-up, attention-matched comparative designs, standardised reporting of exercise dose/intensity fidelity and adverse events, and explicit equity and implementation outcomes before it will be possible to determine whether technology tiers are sufficient for specific patient groups. Overall, the current evidence is most consistent with the view that technology can improve the delivery, monitoring, and personalisation of exercise, but that its clinical value depends on how effectively it is embedded within a structured behaviour-change pathway rather than on the device or platform alone.

## Figures and Tables

**Table 1:** Evidence map of technology-enabled clinical studies relevant to cardiometabolic syndrome (MetS) and adjacent cardiometabolic phenotypes. Studies in MetS-defined cohorts represent direct MetS evidence, whereas studies in MetS risk/prevention, metabolic risk, impaired glucose regulation, prediabetes with overweight/obesity, and type 2 diabetes are included as adjacent translational evidence when outcomes mapped directly onto MetS components or informed physiology-guided exercise personalisation.

Technology	Study (Population)	Design/Duration	Technology Function	Key Outcomes (Reported)
Web-based physical activity intervention	Bosak et al. (MetS) [18]	RCT; 6 weeks	Internet-delivered physical activity intervention	Feasibility/acceptability; intervention engagement; supports web-based delivery of physical activity in MetS
Wearable + app feedback	Huh et al. (MetS) [19]	Pilot; ~12 weeks	Wearable linked to app; feedback/goals	PA engagement; selected MetS components
Wearable + provider feedback	Jang et al. (MetS) [20]	RCT; 12 weeks	Wearable-measured PA + counselling feedback	Waist circumference reduction; improved metabolic components
Mobile + wearable intervention	Kim et al. (MetS risk) [21]	Intervention (observational study)	Mobile + wearable to increase PA	Increased PA; improved health indicators
Apps + wearables (young adults)	Lee et al. (MetS prevention) [22]	Quasi-experimental	Apps + wearables tailored to needs	Improved lifestyle/self-efficacy; ↓ BMI, ↓ cholesterol
Wearable maintenance	Bayerle et al. (employees with MetS) [23]	RCT; maintenance phase	Wearable support after structured exercise	Health outcomes maintained; PA maintenance limited
Telemonitoring-supported PA	Haufe et al. (employees with MetS) [4]	RCT	Remote monitoring + support	↓ MetS severity; improved work ability
Telemonitoring diet + PA	Luley et al. (MetS) [24]	Trial; 12 months	Remote monitoring + feedback	Weight loss; improved MetS markers
mHealth + exercise	Petrella et al. (metabolic risk) [25]	RCT; 12–52 weeks	mHealth support + exercise Rx	Improved BP and risk factors
Mobile care program	Oh et al. (MetS) [26]	RCT	mHealth/SmartCare support	Weight control outcomes
Smart-device lifestyle intervention	Yu et al. (community residents/MetS risk) [15]	Cluster-RCT	Smart-device-based lifestyle self-management with PA component	Improved healthy lifestyle indicators; reduced MetS risk
Individualized mHealth intervention	Chen et al. (MetS) [16]	Quasi-experimental	WeChat mini program + individualized follow-up	Improved physical activity behavior; reduced cardiovascular risk
IVRS-based mHealth	Sharma et al. (MetS) [17]	Cluster randomized trial	Interactive voice response system + mHealth follow-up	Reduced cardiovascular risk factors in MetS
AI coaching	Mathioudakis et al. (prediabetes + overweight/obesity) [27]	RCT	Fully automated AI-DPP vs. human	Noninferior composite: weight, HbA1c, PA
VR exergame	Mologne et al. (adults) [28]	RCT; 12 weeks	Immersive VR + adaptive resistance	Improved fitness; improved cardiometabolic measures
Exergaming	Wu et al. (MetS) [29]	RCT	Exergame-based exercise	Improved executive function; feasibility
CGM for adherence	Bailey et al. (impaired glucose) [30]	Pilot RCT	Real-time CGM feedback	Improved exercise adherence
CGM-personalised timing	Chang et al. (T2D) [31]	RCT; 8 weeks	Exercise timed to glycemic patterns	Improved peak glucose; vascular outcomes
CGM ± tracker	Lahiri et al. (T2D) [32]	RCT	Sequential CGM with/without tracker	Improved glycemic control

Abbreviations: MetS, metabolic syndrome; PA, physical activity; RCT, randomized controlled trial; mHealth, mobile health; AI, artificial intelligence; DPP, Diabetes Prevention Program; VR, virtual reality; CGM, continuous glucose monitoring; T2D, type 2 diabetes; BP, blood pressure; Rx, prescription.

## Data Availability

No new data were created in this study. All information supporting the findings of this study is contained within the article and its Supplementary Materials.

## Supplementary Materials

The following supporting information can be downloaded at https://www.mdpi.com/article/10.3390/jfmk11020153/s1. Table S1: PRISMA-ScR checklist; Figure S1: PRISMA-ScR flow diagram; Supplementary Material S1: data-base-specific search strings and yields.
