# Supplementary material for: Technology-Enhanced Exercise Training for Cardiometabolic Syndrome: A Scoping Review
Source: J Funct Morphol Kinesiol. 2026 Apr 14;11(2):153. doi: 10.3390/jfmk11020153 (PMC13108096; doi:10.3390/jfmk11020153)
Supplement: Supplementary file 1 [file jfmk-11-00153-s001.zip › jfmk-4204278-supplementary.pdf]

Journal of
Functional Morphology and Kinesiology

MDPI

Supplementary Materials

This document includes:

• Table S1: PRISMA-ScR checklist.

• Figure S1: PRISMA-ScR flow diagram.

• Supplemenary Materials S1: Database-specific search strategies for PubMed, Scopusand Web of Sciencetogether with details of the supplementary IEEE Xplore search and post hoc supplementary Embase check.

Supplemenary Materials S1. Database Search Strategy (PubMed, Scopus, and Web of Science; supplementary IEEE Xplore search; and post hoc supplementary Embase check). This appendix provides the database-specific search documentation for the present scoping review and accompanies the PRISMA-ScR reporting materials provided in Table S1 and Figure S1.

Searches were conducted using combinations of terms capturing (i) cardiometabolic phenotypes (metabolic syndrome/cardiometabolic; and selected adjacent phenotypes where relevant), (ii) exercise/physical activity interventions, and (iii) technology enablers (wearables, telemonitoring/mHealth, AI coaching, VR/exergaming, and continuous glucose monitoring).

Date searched: PubMed: January 12-15, 2026

Scopus, Web of Science, and the supplementary IEEE Xplore search: March 2026

Post hoc supplementary Embase check: during manuscript refinement

The full PubMed search strategy is presented below; equivalent database-adapted searches were used in Scopus and Web of Science, and a concept-aligned supplementary search was performed in IEEE Xplore.

PubMed

Q1 (MetS/cardiometabolic + exercise/PA + wearables/apps/telemonitoring/mHealth): n = 282

(("metabolic syndrome"[Title/Abstract] OR "cardiometabolic syndrome"[Title/Abstract]) AND (exercise[Title/Abstract] OR "exercise training"[Title/Abstract] OR "physical activity"[Title/Abstract] OR training[Title/Abstract]) AND (wearable*[Title/Abstract] OR smartwatch*[Title/Abstract] OR "activity tracker*"[Title/Abstract] OR acceleromet*[Title/Abstract] OR telemonitor*[Title/Abstract] OR "remote monitor*"[Title/Abstract] OR mhealth[Title/Abstract] OR "mobile health"[Title/Abstract] OR app[Title/Abstract] OR smartphone[Title/Abstract])) NOT (review[Publication Type] OR protocol[Title] OR "case reports"[Publication Type])

Q2 (MetS/cardiometabolic + exercise/PA + VR/exergaming): n = 7

(("metabolic syndrome"[Title/Abstract] OR "cardiometabolic syndrome"[Title/Abstract]) AND (exercise[Title/Abstract] OR "physical activity"[Title/Abstract] OR training[Title/Abstract]) AND ("virtual reality"[Title/Abstract] OR VR[Title/Abstract] OR exergam*[Title/Abstract] OR "serious game*"[Title/Abstract])) NOT (review[Publication Type] OR protocol[Title] OR "case reports"[Publication Type])

Q3 (AI coaching + exercise/PA + intervention terms; cardiometabolic phenotypes): n = 144

((prediabet*[Title/Abstract] OR "type 2 diabetes"[Title/Abstract] OR overweight[Title/Abstract] OR obesity[Title/Abstract] OR "metabolic syndrome"[Title/Abstract] OR cardiometabolic[Title/Abstract]) AND (exercise[Title/Abstract] OR "physical activity"[Title/Abstract]) AND ("artificial intelligence"[Title/Abstract] OR "AI-powered"[Title/Abstract] OR "machine learning"[Title/Abstract] OR chatbot*[Title/Abstract] OR "digital coach*"[Title/Abstract] OR "automated coach*"[Title/Abstract]) AND (random*[Title/Abstract] OR trial[Title/Abstract] OR intervention*[Title/Abstract])) NOT (review[Publication Type] OR protocol[Title] OR "case reports"[Publication Type])

Q4 (CGM + exercise/PA + intervention terms; dysglycaemia/cardiometabolic phenotypes): n = 126

(("continuous glucose monitor*"[Title/Abstract] OR CGM[Title/Abstract]) AND (exercise[Title/Abstract] OR "exercise training"[Title/Abstract] OR "physical activity"[Title/Abstract]) AND (randomized[Title/Abstract] OR randomised[Title/Abstract] OR "randomized controlled trial"[Title/Abstract] OR trial[Title/Abstract] OR intervention*[Title/Abstract]) AND ("type 2 diabetes"[Title/Abstract] OR T2D[Title/Abstract] OR prediabet*[Title/Abstract] OR "impaired glucose"[Title/Abstract] OR "metabolic syndrome"[Title/Abstract] OR cardiometabolic[Title/Abstract])) NOT (review[Publication Type] OR protocol[Title] OR "case reports"[Publication Type])

Q5 (Metabolic Syndrome [MeSH] + exercise/PA + technology terms): n = 23

(("Metabolic Syndrome"[MeSH Terms]) AND (exercise[Title/Abstract] OR "exercise training"[Title/Abstract] OR "physical activity"[Title/Abstract]) AND (wearable*[Title/Abstract] OR telemonitor*[Title/Abstract] OR mhealth[Title/Abstract] OR "mobile health"[Title/Abstract] OR "virtual reality"[Title/Abstract] OR exergam*[Title/Abstract] OR "continuous glucose monitor*"[Title/Abstract] OR CGM[Title/Abstract] OR "artificial intelligence"[Title/Abstract] OR "digital coach*"[Title/Abstract])) NOT (review[Publication Type] OR protocol[Title] OR "case reports"[Publication Type])

Across the primary databases, the initial yields were PubMed, n = 582; Scopus, n = 27; and Web of Science, n = 29. A supplementary IEEE Xplore search was also conducted using concept blocks aligned to the main search strategy (cardiometabolic phenotype, exercise/physical activity, and enabling technology), identifying n = 33 records. After screening, no additional eligible clinical intervention studies were included from this source. Records were exported to a reference manager for cross-database deduplication prior to title/abstract screening and full-text assessment. Final post-deduplication counts are reported in Figure S1. The search expansion beyond PubMed was undertaken to reduce database bias and improve capture of technology-oriented interventions that may not be consistently retrieved in a single biomedical database.

In addition, a post hoc supplementary Embase check was undertaken during manuscript refinement after institutional access limitations had initially prevented Embase from being incorporated into the primary search workflow. This supplementary check identified one additional eligible study (Bosak et al. 2010), which was assessed against the prespecified eligibility criteria and incorporated into the final evidence map. This step is reported separately from the primary database yields above.

Figure S1. PRISMA-ScR Flow Diagram
Identification

Records identified from PubMed (n = 582)
Records identified from Scopus (n = 27)
Records identified from Web of Science (n = 29)
Total records identified from primary databases (n = 638)

↓

Records removed before screening as duplicates across databases (n = 325)

↓

Records screened after deduplication (n = 313)
→Records excluded after title/abstract screening (n = 203)

↓

Full-text reports sought for retrieval (n = 110)
→ Full-text reports not retrieved (n = 0)

↓

Full-text reports assessed for eligibility from the primary workflow (n = 110)
→ Full-text reports excluded from the primary workflow (n = 92)

↓

Studies included from the primary workflow (n = 18)

+

Additional eligible study identified through post hoc supplementary Embase check during manuscript refinement (n = 1)

↓

Total studies included in the scoping review (n = 19)

Note: Records were identified through PubMed, Scopus, and Web of Science and deduplicated across databases before screening. A supplementary search of IEEE Xplore was conducted to improve the capture of technology-oriented literature; however, no additional eligible clinical intervention studies were identified from this source. During manuscript refinement, a post hoc supplementary Embase check identified one additional eligible study (Bosak et al. 2010), which was assessed against the prespecified eligibility criteria and incorporated into the final evidence map. Only the final, internally coherent PRISMA-ScR workflow is presented here; superseded draft counts and earlier single-database flow summaries have been removed from the supplementary materials.

Table S1. PRISMA-ScR Checklist

Section	PRISMA-ScR item	Where addressed
Title	Identify the report as a scoping review.	Title page
Abstract	Provide structured summary.	Abstract
Introduction—Rationale	Describe the rationale for the review.	Introduction
Introduction—Objectives	Provide an explicit statement of the questions and objectives.	Introduction (end)
Methods—Protocol/registration	Indicate whether a review protocol exists and any registration information (if applicable).	Methods (Protocol/registration statement)
Methods—Eligibility criteria	Specify characteristics of sources of evidence and report characteristics used as eligibility criteria.	Methods (Eligibility criteria)
Methods—Information sources	Describe all information sources (e.g., databases) and date of the most recent search.	Methods + Appendix S1 (PubMed, Scopus, and Web of Science; supplementary IEEE Xplore search; and post hoc supplementary Embase check)
Methods—Search	Present full electronic search strategy for at least one database.	Appendix S1 (full PubMed strategy; database-adapted equivalent searches for Scopus and Web of Science; supplementary IEEE Xplore search described; post hoc supplementary Embase check described)
Methods—Selection of sources of evidence	Describe the process for selecting sources of evidence.	Methods (Screening) + Figure S1
Methods—Data charting process	Describe the methods of charting data from included sources of evidence.	Methods (Data charting)
Methods—Data items	List and define all variables for which data were sought.	Methods (Outcomes charted) + Table 1

Methods—Critical appraisal	If done, provide a rationale and describe methods; if not done, state so.	Methods (Critical appraisal: not performed)
Results—Selection of sources of evidence	Give numbers of sources screened, assessed, and included, with reasons for exclusions.	Figure S1 + Results
Results—Characteristics of sources of evidence	Present characteristics for which data were charted.	Table 1
Results—Critical appraisal within sources	If done, present data; otherwise not applicable.	Not applicable
Results—Results of individual sources	Present the relevant results charted from each source.	Results + Table 1
Discussion—Summary of evidence	Summarize the main results, including an overview of concepts, themes, and evidence types.	Discussion
Discussion—Limitations	Discuss limitations of the scoping review process.	Discussion (Limitations)
Conclusions	Provide a general interpretation and implications for future research.	Conclusions
Funding	Describe sources of funding for the included evidence and for the review.	Funding statement
